# Intraspecific diversity of *Staphylococcus aureus* populations isolated from cystic fibrosis respiratory infections

**DOI:** 10.1128/spectrum.00367-26

**Published:** 2026-07-06

**Authors:** Ashley M. Alexander, Hui Qi Loo, Lauren Askew, Vishnu Raghuram, Sarah W. Satola, Timothy D. Read, Joanna B. Goldberg

**Affiliations:** 1 Population Biology, Ecology, and Evolution Program, Graduate Division of Biological Sciences, Laney Graduate School, Emory University, Atlanta, Georgia, USA; 2 Department of Biology, College of Arts and Sciences, Georgia State University, Atlanta, Georgia, USA; 3 Department of Quantitative Biomedicine, University of Zürich, Zürich, Switzerland; 4 Department of Biology, Emory University, Atlanta, Georgia, USA; 5 Biochemistry, Cell and Developmental Biology Program, Graduate Division of Biological and Biomedical Sciences, Laney Graduate School, Emory University 1371, Atlanta, Georgia, USA; 6 Microbiology and Molecular Genetics Program, Graduate Division of Biological and Biomedical Sciences, Laney Graduate School, Emory University 1371, Atlanta, Georgia, USA; 7 Division of Infectious Diseases, Department of Medicine, Emory University School of Medicine, Atlanta, Georgia, USA; 8 Department of Pediatrics, Division of Pulmonary, Asthma, Cystic Fibrosis, and Sleep, Emory University School of Medicine, Atlanta, Georgia, USA; LSU Health Shreveport, Shreveport, Louisiana, USA

**Keywords:** cystic fibrosis, *Staphylococcus aureus*, intraspecific diversity, microbial ecology and evolution

## Abstract

Long-term associations within an individual host allow bacterial populations to diversify over time, sometimes resulting in the coexistence of multiple species or variants of the same species. Traditional methods for identifying infective agents generally involve isolating a single pathogen and potentially even a single colony from a given sample. While screening samples for virulent or difficult-to-treat pathogens is an important part of informing clinical treatment and correlative research, these reductive methods alone do not provide researchers or healthcare providers with the potentially important perspective on the true pathogen population. In this study, we begin to address this limitation by comparing the phenotypic and genotypic diversity of single colonies and pooled isolates of *Staphylococcus aureus* taken from fresh sputum samples from three patients with cystic fibrosis. Additionally, we compare the results collected in our research laboratory to those processed by the Emory Clinical Microbiology Laboratory with the identical sputum samples. We identified unrelated coexisting lineages in two out of three sputum samples, as well as clinically relevant population structures that were not apparent from studying single-colony isolates alone. Altogether, our observations presented here demonstrate that the true nature and phenotype of a clinically isolated pathogen can be missed with standard sampling methods when assessing chronic infections. More broadly, this work outlines the potential impact that comprehensive population-level sampling may have for both research efforts and more effective treatment practices.

**IMPORTANCE:**

When obtaining bacterial isolates from infections, it is important to consider the ecological biases introduced by methods used for collection and processing. In this study, we demonstrate how reductive sampling and processing methods traditionally used by clinical microbiology labs often do not adequately capture complex and clinically relevant traits present in diverse pathogen populations. When treating or studying bacteria like *Staphylococcus aureus* that can maintain multiple variants within a population over a long period of time, it may be more effective and informative to employ sampling methods that account for the potential diversity within a population, as outlined in our approach presented here.

## INTRODUCTION

*Staphylococcus aureus* became the most common respiratory pathogen infecting individuals with the genetic disease, cystic fibrosis (CF), in the early 2000s, surpassing *Pseudomonas aeruginosa* (1). Like many opportunistic pathogens, *S. aureus* is a species with significant diversity across lineages (2–4), and it has been observed that mixed populations of genetically distinct *S. aureus* strains can co-occur within an individual host (5–7). However, current clinical surveillance techniques generally do not allow for the detection of multiple lineages of *S. aureus* from people with CF (pwCF), as usually only one representative colony is isolated from the respiratory sample (7). While these methods are generally considered the “gold standard” and are useful for identifying highly prevalent pathogens that may be difficult to treat, they are likely not reflective of the true population structure—or the number and frequency of coexisting lineages within a complex chronic infection. In the case of *S. aureus* in particular, clinical processing is heavily focused on identifying whether MRSA (methicillin-resistant *Staphylococcus aureus*) is present in a sample. While this observation is undoubtedly important for informing treatment practices and research alike, it is possible that the focus on resistant populations masks the potential identification of a lineage that may be responsible for the pathology and disease being treated.

To begin to address this gap in surveillance and understanding, we present an in-depth look at the diversity within populations of *S. aureus* isolated from fresh sputum collected from three different pwCF by sampling six to eight single colonies and one to two whole-population pool samples from each sputum sample. We compare our phenotypic and genotypic analysis of these populations with those of the identical sputum samples processed by the clinical microbiology laboratory. For two of three samples, we were able to isolate and identify more than one unrelated lineage that was present in the *S. aureus* population of the sampled sputum. In the context of the observed phenotypes, our findings show that intraspecific diversity can have significant clinical implications when it comes to traits related to disease severity and treatment potential, including antimicrobial resistance, hemolysis, and quorum sensing activity. Our observations discussed in this study contribute to a growing body of research exploring the intraspecific diversity that exists within individual patients and typical population structures of CF-associated pathogens (4, 8). Considering sampling methods that account for and quantify intraspecific diversity in clinical samples may assist in the design of targeted treatment plans for individuals with chronic infections and would inform continued research on population structure and dynamics in chronic infections.

## RESULTS

### Clinical sample analysis

**Fig 1:**
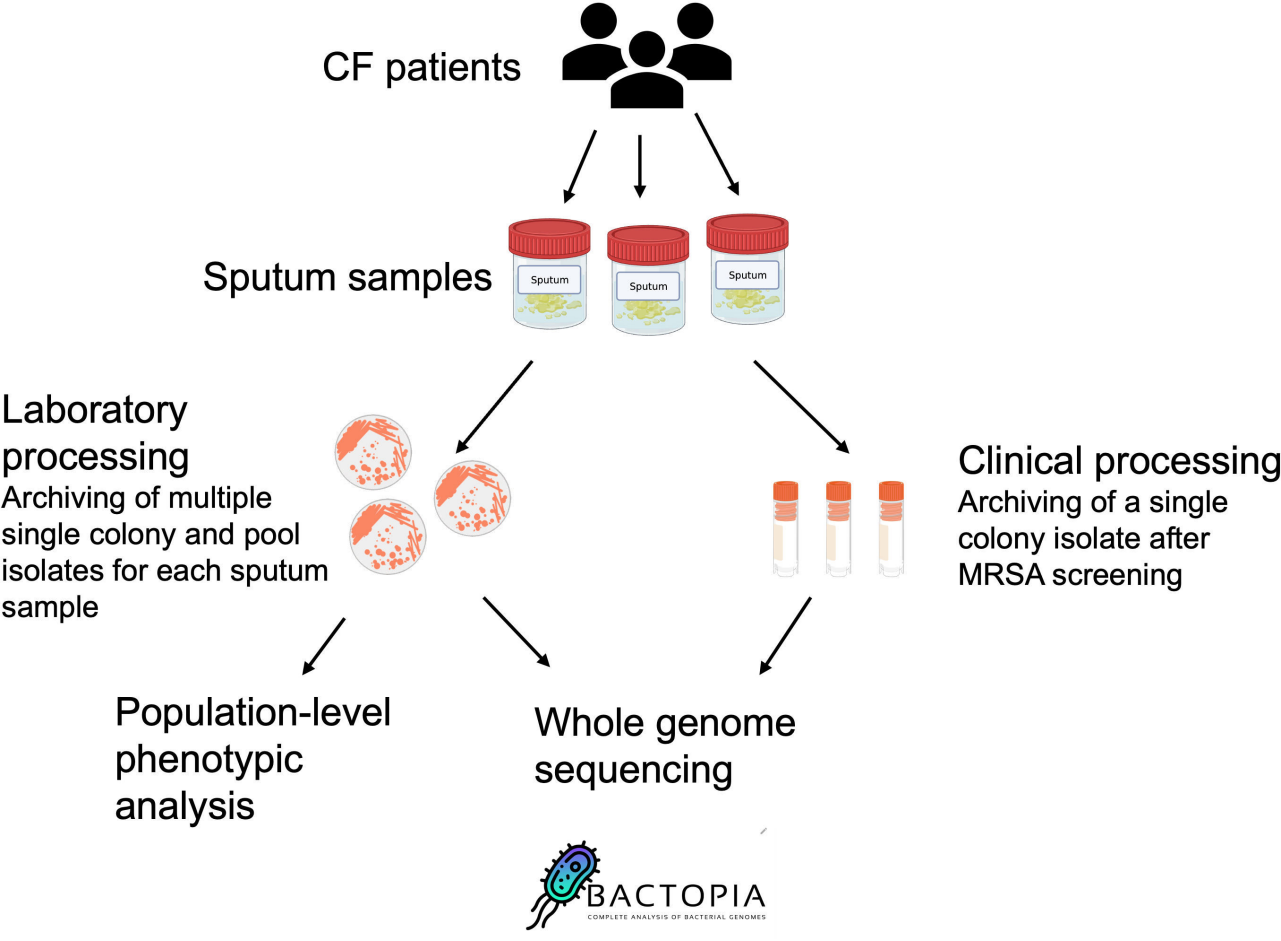
Sampling methods. Method for processing *S. aureus* isolates from three patients’ fresh sputum samples by the Goldberg lab and the Emory Clinical Microbiology Laboratory. During laboratory processing, eight single-colony isolates were archived per patient sample, and one to two pool samples were taken for each sputum sample by scraping all remaining colonies. Isolates and pools were then phenotypically and genotypically analyzed for their diversity of traits as described in the text. Whole-genome sequences were analyzed using the bioinformatics pipeline Bactopia v3.0.1 (9). Graphics were created with BioRender.

Expectorated sputum was collected from three different pwCF, and identical samples were processed, analyzed, and archived by both the Goldberg lab and the Emory Clinical Microbiology Laboratory (Fig. 1). At collection, the Emory Clinical Microbiology Laboratory performed antibiotic susceptibility testing on single-colony isolates from Patient 1 and Patient 2. Patient 1’s clinically isolated *S. aureus* was identified as being resistant to clindamycin and susceptible to oxacillin, tetracycline, trimethoprim, sulfamethoxazole, and vancomycin. Clinically isolated *S. aureus* from Patient 2 was identified as being resistant to clindamycin as well as oxacillin and susceptible to daptomycin, linezolid, tetracycline, trimethoprim, sulfamethoxazole, and vancomycin (Table 1). Oxacillin was used by the Emory Clinical Microbiology Laboratory to determine beta-lactam resistance and MRSA status of an *S. aureus* sample. Patient 3’s sputum sample was taken shortly before the Emory Clinical Microbiology Laboratory was closed due to the COVID-19 pandemic; therefore, they were unable to process this sample for antibiotic susceptibilities. The archived clinically isolated samples were obtained and tested for hemolysis activity; only Patient 1’s *S. aureus* clinical isolate was positive for alpha hemolysis. Whole-genome sequencing was also conducted on all three isolates (1_CFBR, 2_CFBR, and 3_CFBR). Bactopia was used to determine the multilocus sequence types (MLST) for all three samples as well as *agr* group (Table 1).

**TABLE 1:** Antibiotic resistance report of *S. aureus* isolated from the corresponding CF patients from the Emory Clinical Microbiology Laboratory

Patient (sample name)	Antibiotic susceptibility*^a^*							CFBR ID	Sample date	Hemolysis	Sequence type	*agr* group
	Clindamycin	Daptomycin	Linezolid	Oxacillin^*b*^	Tetracycline	Trimethoprim/sulfamethoxazole	Vancomycin					
1 (1_CFBR)	Resistant	NA	NA	Susceptible	Susceptible	Susceptible	Susceptible	623	2/20/2020	+	72	1
2 (2_CFBR)	Resistant	Susceptible	Susceptible	Resistant	Susceptible	Susceptible	Susceptible	196	3/12/2020	−	225	2(f)
3 (3_CFBR)	NA	NA	NA	NA	NA	NA	NA	311	3/12/2020	−	5	2(f)

^*a*^ *NA*: susceptibility test was not performed by the clinical microbiology laboratory. *agr* group (f) indicates that a frameshift mutation was detected.

^*b*^ + indicates the presence of hemolytic activity; − indicates the absence of hemolytic activity.

### Phenotypic analysis of populations

To assess the phenotypic diversity of all single colony and pool isolates processed in the Goldberg lab, within and between patients, we first started by assessing the colony morphology of all samples on TSA plates. At initial processing, two small colony variants were identified from Patient 1’s MSA plate; however, we were unable to revive these isolates on SIA or brain-heart-infusion (BHI) agar, even after several days of incubation on rich agar and additional supplementation with amino acids. All other isolates from all three patients appeared very similar to each other in colony size and texture. Notably, colony color varied across isolates. Patient 1’s isolates were all white in appearance on SIA plates, while all Patient 2’s isolates were yellow. Patient 3’s single-colony isolates were yellow, while the pool sample 3_110_Pool had both white and yellow colonies, suggesting that there is an additional population (white) that is not represented in any single-colony isolate from this patient (Table 2).

**TABLE 2:** Compilation of genotypes and phenotypes of single colony and pool isolates of *S. aureus* from CF sputum^*a*^

Patient	Isolate ID	Isolate type	Sequence type	*agr* group	MRSA status by ChromAgar	Colony color	Alpha toxin hemolysis	Polysaccharide production on Congo Red
1 (CFBR ID: 623)	1_90_Pool	Pool	72	1	S	White	+	Red/matte (overproducer)
	1_91_Pool	Pool	72	1	S	White	+	Red/matte (overproducer)
	1_92_SC	Single colony	72	1	S	White	+	Red/matte (overproducer)
	1_93_SC	Single colony	72	1	S	White	+	Red/matte (overproducer)
	1_94_SC	Single colony	72	1	S	White	+	Red/matte (overproducer)
	1_95_SC	Single colony	72	1	S	White	+	Red/matte (overproducer)
	1_96_SC	Single colony	72	1	S	White	+	Red/matte (overproducer)
	1_97_SC	Single colony	72	1	S	White	+	Red/matte (overproducer)
2 (CFBR ID: 196)	2_100_Pool	Pool	5^*a*^ 6/7	2	R	Yellow	+	Red/matte (overproducer)
	2_101_SC	Single colony	5^*a*^ 6/7	2	S	Yellow	+	Red/matte (overproducer)
	2_102_SC	Single colony	5^*a*^ 6/7	2	S	Yellow	+	Red/matte (overproducer)
	2_103_SC	Single colony	5^*a*^ 6/7	2	S	Yellow	+	Red/matte (overproducer)
	2_104_SC	Single colony	5^*a*^ 6/7	2	S	Yellow	+	Red/matte (overproducer)
	2_105_Pool	Pool	5^*a*^ 6/7	2	R	Yellow	+	Red/matte (overproducer)
	2_105_MRSA	ChromAgar Isolation	225	2(f)	R		−	
	2_106_SC	Single colony	5^*a*^ 6/7	2	S	Yellow	+	Red/matte (overproducer)
	2_107_SC	Single colony	5^*a*^ 6/7	2	S	Yellow	+	Red/matte (overproducer)
	2_108_SC	Single colony	5^*a*^ 6/7	2	S	Yellow	+	Red/matte (overproducer)
	2_109_SC	Single colony	5^*a*^ 6/7	2	S	Yellow	+	Red/matte (overproducer)
3 (CFBR ID: 311)	3_110_Pool	Pool	5^*a*^ 4/7	1/2(m)	R	Yellow and White	+	Red/mixed shiny and matte (normal/nonproducer)
	3_111_SC	Single colony	5	2(f)	R	Yellow	−	Red/shiny (nonproducer)
	3_112_SC	Single colony	5	2(f)	R	Yellow	−	Red/shiny (nonproducer)
	3_113_SC	Single colony	5	2(f)	R	Yellow	−	Dark/matte colonies (normal)
	3_114_SC	Single colony	5	2(f)	R	Yellow	−	Red/matte and shiny (normal/overproducer)
	3_115_SC	Single colony	5	2(f)	R	Yellow	−	Red/shiny (nonproducer)
	3_116_SC	Single colony	5	2(f)	R	Yellow	−	Red/shiny (nonproducer)
	3_117_SC	Single colony	8	1	S	Yellow	+	Red/matte (overproducer)
	3_118_SC	Single colony	8	1	S	Yellow	+	Red/matte (overproducer)

^*a*^ Not a complete match; fraction after indicates the number of loci that map to that ST out of seven total loci used to assign ST; +, clear hemolysis; −, no hemolysis. (m) indicates that multiple *agr* groups were detected. (f) indicates that a frameshift mutation was identified in the *agrC *locus. MRSA status based on ChromAgar is denoted as resistant (R) and sensitive (S).

All isolates from Patients 1 and 2 (both pool and single colonies) were positive for alpha toxin hemolysis when cultured on sheep blood agar. For Patient 2, these results did not align with the corresponding clinical isolate (2_CFBR) that we obtained from the Emory Clinical Microbiology Laboratory, which was negative for hemolysis. In Patient 3, only the pool sample and isolates 3_117_SC and 3_118_SC were positive for alpha hemolysis (Table 2); this also was distinct from what we observed for the single colony, 3_CFBR, obtained from the Emory Clinical Microbiology Laboratory.

All isolates from Patients 1 and 2 (both pools and single colonies) were determined to be overproducers of polysaccharide based on their rough and matte appearance on Congo Red Agar (CRA) (2). Isolates from Patient 3 appeared more heterogeneous. Both the patient pool sample and single-colony isolate samples presented with some variation on CRA, with isolates 3_110_Pool and 3_114_SC having both textured and shiny red colonies and therefore classified as mixtures of normal and overproducing cells. Isolate 3_113_SC appeared as dark shiny colonies, classifying it as a normal producer based on the darker color. Isolates 3_115_SC and 3_116_SC were bright red and shiny on plates, classifying them as clear nonproducers. Finally, isolates 3_117_SC and 3_118_SC from Patient 3 were determined to be overproducers based on their red and matte appearance on CRA (Table 2).

ChromAgar plates were used as an additional test of MRSA status. Colony growth with purple pigmentation on ChromAgar can be reliably used to detect methicillin resistance in *S. aureus*, as described in Samra et al. (10). All isolates from Patient 1 showed no growth on ChromAgar plates and therefore were determined to be MSSA. All single-colony isolates from Patient 2 were determined to be MSSA (methicillin-sensitive *Staphylococcus aureus*); however, both pool samples showed some pigmented colonies on ChromAgar plates. The MRSA subpopulation isolated on ChromAgar was archived and later included in genomic analyses as sample 2_105_MRSA. Single-colony isolates from Patient 3, 3_111_SC, 3_112_SC, 3_113_SC, 3_114_SC, 3_115_SC, and 3_116_SC, as well as the pool sample 3_110_Pool, were determined to be MRSA, with pigmented colony growth observed for all of the above samples. Samples 3_117_SC and 3_118_SC from the same patient were determined to be MSSA based on their lack of growth on ChromAgar (Table 2).

### Genotypic analysis

Whole-genome sequence data were successfully obtained for all isolates. Genomes were assembled and processed using the Bactopia pipeline. Multilocus sequence type (MLST) was determined for each genome using the MLST tool included in the Bactopia pipeline, which for *S. aureus* includes variable alleles across seven loci. Single-colony isolates from Patient 2 and the pool sample from Patient 3 did not match any known MLST types; therefore, the output is listed as the closest matching MLST with the number of matching sites out of seven indicated (Table 2).

**Fig 2:**
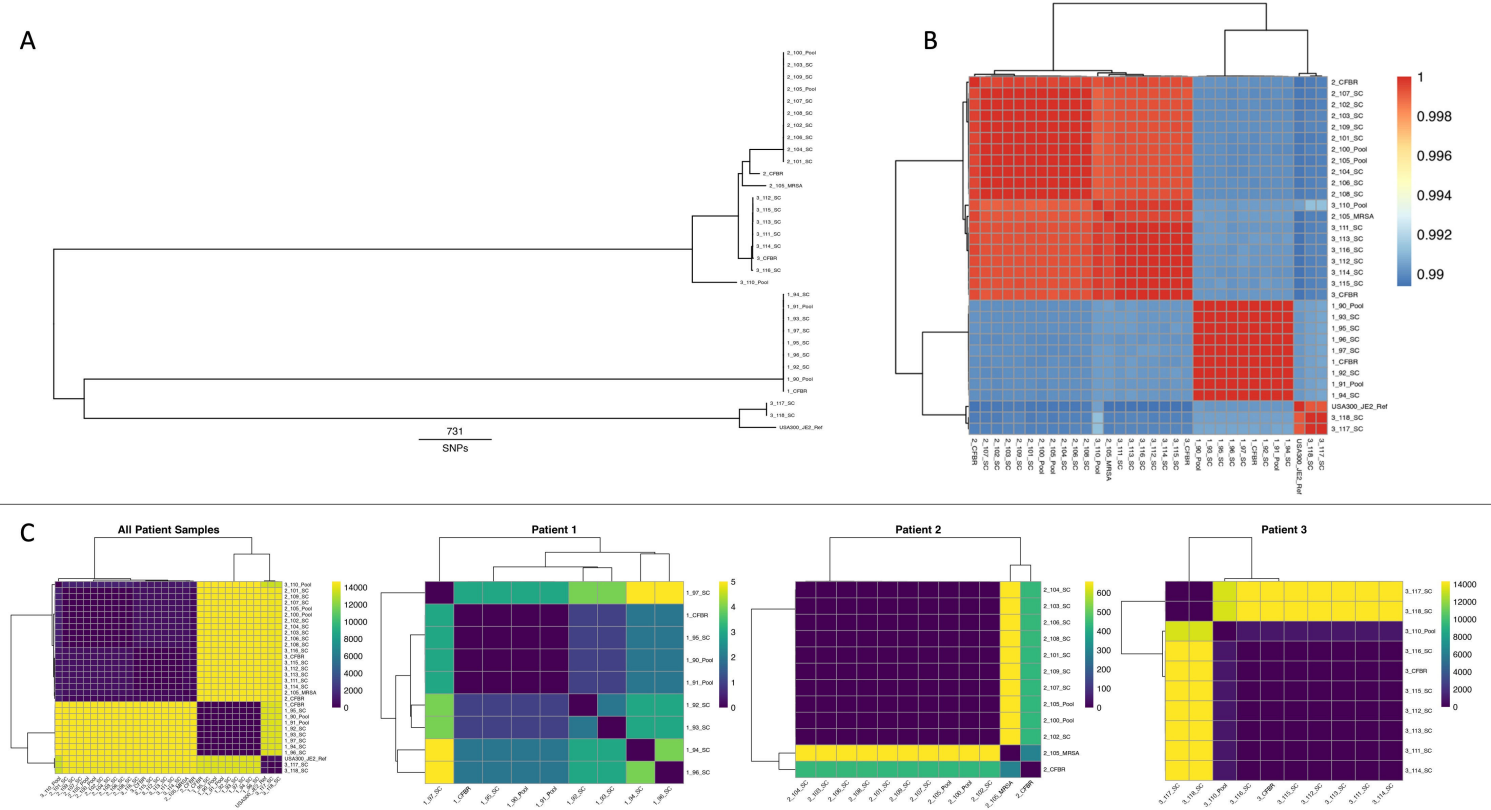
Genetic distances across all patient isolates. (**A**) Phylogenetic tree of isolates based on their SNP distance calculated from the Parsnp genome alignments with USA 300 JE2 used as a reference and rooted at the midpoint of the sample set. SNP distance ranges from 0 to 14,500 SNPs across all patient samples. Pooled samples are included as consensus genomes to illustrate their relatedness to other samples in the set. (**B**) Heatmap generated with the R package pheatmap visualizing the ANI between all patient samples, which was calculated using the Python package pyANI. Scale is based on ANI difference (1 = 100% identical sequences). Pooled samples are also included in this analysis as consensus genomes, illustrating the relatedness of the majority populations in the pools. (**C**) Heatmaps of SNP distances across and within patient sample sets. The maximum SNP distance within Patient 1 samples is 5; for Patient 2, the maximum number of SNPs is 658; and for Patient 3, it is 14,325. For individual patient heatmaps, the reference genome USA 300 JE2 was excluded to enable appropriate resolutions for each sample set.

To determine isolate relatedness, assemblies were aligned with Parsnp using USA300_JE2, NCBI accession number NC_007793, as a reference strain (9, 11, 12). Parsnp output was then used to create a phylogenetic tree based on single-nucleotide polymorphism (SNP) distances, using the tool Disty to create a distance matrix and the R packages ape and ggtree to visualize this tree (13, 14) (Fig. 2A). The resulting phylogeny illustrates how samples from individual pwCF generally cluster closely together. However, there were exceptions, including 3_117_SC, 3_118_SC, and 3_110_Pool, which appear to cluster separately from other isolates from Patient 3. Additionally, the subpopulation identified in the ChromAgar analysis for Patient 2, 2_105_MRSA, and the corresponding clinical isolate 2_CFBR cluster separately from other Patient 2 isolates. These exceptions reflect other phenotypic and genotypic findings. In the case of Patient 3, samples 3_117_SC and 3_118_SC had differences in their MRSA status, *agr* group, sequence type, and hemolysis compared to other single-colony isolates and the pool sample 3_110_P. The pool sample from Patient 3 likely contained sequence data from both detected populations (MSSA and MRSA) and potentially other undetected populations, and therefore did not cluster with either group of single-colony isolates. For Patient 2, isolate 2_105_MRSA shared some genotypic similarities with the clinical sample 2_CFBR, including its methicillin resistance status; however, these two samples are not part of a monophyletic group. For Patients 1 and 3, the corresponding clinical samples, 1_CFBR and 3_CFBR, clustered with the majority of single-colony isolates from their respective patient sample sets. Samples CFBR_2 and the control reference strain, USA_300_JE2, had the lowest ANI value of 98.94% and several single-colony isolates from within the same patient were identified as 100% identical. Among Patient 1 samples, 1_95_SC, 1_91_Pool, and 1_90_Pool were all identical, with 1_95_SC and 1_91_Pool also being identical to 1_CFBR. From Patient 2, samples 2_100_Pool and 2_107_SC, 2_105_Pool and 2_102_Pool, and 2_109_SC and 2_102_SC, all were identical based on ANI. From Patient 3, 3_112_SC was identical to 3_115_SC and 3_113_SC. Overall, ANI analysis confirmed that isolates from Patient 1 are genetically identical. Based on ANI, all Patient 2 samples clustered closely together, including the pool samples, again with the exception of the CFBR_2 isolate and the 2_105_MRSA sample. When comparing ANI across isolates from Patient 3, samples 3_117_SC and 3_118_SC cluster closely to the reference strain USA300_JE2_Ref but were distant from other Patient 3 isolates and distant from other isolates across the sample set. The pool sample from Patient 3, 3_110_Pool, did not cluster with either set of single-colony isolates from Patient 3 (Fig. 2B). To further visualize the SNP distances within and between patient samples, heatmaps for all samples and each patient sample set are shown in Fig. 2C. Patient 1 has the lowest range of SNP differences among its samples, with five being the maximum number of SNPs between isolates. Patient 2 has a moderate level of SNP differences, with the MRSA isolate 2_105_MRSA and the isolate processed by the clinical lab, 2_CFBR, having the greatest SNP distances compared to the rest of the isolates, and the maximum SNP distance occurring between 2_105_MRSA and 2_106_SC, with 658 SNPs. Patient 3 had the greatest SNP distances within its sample set, with the maximum number of SNPs between samples at 14,325. Notably, samples 3_117_SC and 3_118_SC were found to have no SNPs in their core genome alignment.

In this analysis of sample relatedness (Fig. 2A through C), pooled samples are included, but we note here an important distinction: these samples are treated as consensus genomes representing the majority population present in their sequences. Pooled samples will likely contain a high number of minor alleles, and therefore, accounting for the exact number of single-nucleotide variants is not possible for these samples. We find it useful, however, to visualize their general relatedness to other samples across and within their patient sample sets, as this yields some important insights as to what can be observed with this type of sampling method.

**Fig 3:**
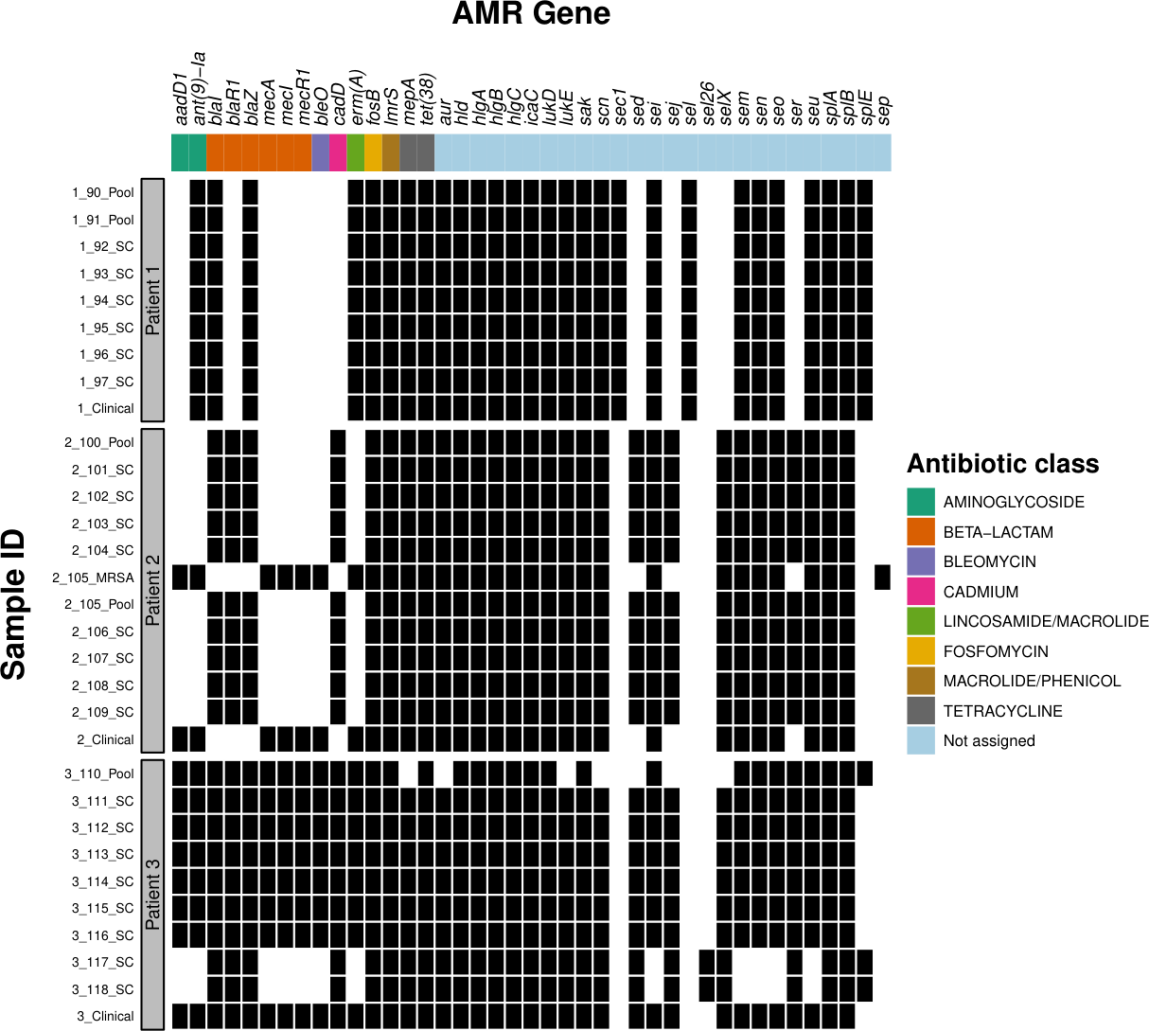
Antimicrobial resistance genetic signatures. Black boxes indicate the presence of alleles associated with antimicrobial resistance, as identified by the AMRFinderPlus tool included in the Bactopia pipeline. Colors across the top of the figure group genes by the antibiotic class with which their resistance is associated.

Genes associated with antibiotic resistance were identified in the genomic data for all isolates using the NCBI AMRFinderPlus program as part of the Bactopia genomic analysis pipeline (9, 15) (Fig. 3). Patient 1’s isolates all shared the same antibiotic resistance gene profiles. All single colony and pool isolates from Patient 2 had identical antibiotic resistance gene profiles, while the isolates obtained from the Emory Clinical Microbiology Laboratory (2_CFBR) and 2_105_MRSA were very similar to one another, with the exception of the resistant *sep* allele being present in 2_105_MRSA. All isolates in the entire sample set from all three patients had alleles associated with resistance to fosfomycin (*fosB*) and tetracycline (*mepA* and *tet38*). Methicillin resistance alleles (*mecA*, *mec1*, and *mecR1*) were not present in single colony and pool isolates from Patients 1 and 2, but variants of *blaI* and *blaZ*, associated with beta-lactam resistance, were present in these samples, with the addition of *blaR1* only in Patient 2 samples. Additionally, all isolates from Patient 1 had alleles *aadD1* and *erm(A*), associated with resistance to aminoglycosides and erythromycin. All alleles associated with antibiotic resistance in the NCBI AMRFinderPlus database, with the exception of *sed*, *sel26*, *seIX, splE,* and *sep,* but including all *mec* alleles associated with methicillin resistance, were identified as present in the genomes of all single colonies from Patient 3, with the exception of samples 3_117_SC and 3_118_SC. Isolates 3_117_SC and 3_118_SC had genetic signatures associated with fosfomycin, tetracycline, and the three *bla* beta-lactam resistance alleles in *blaI, blaR1,* and *blaZ,* among antibiotic resistance genes assigned to an antibiotic class. The pooled sample from Patient 3 has a unique antibiotic resistance gene profile compared to either group of single-colony isolates—notably missing the *mepA* allele associated with tetracycline resistance, along with several alleles not assigned to an antibiotic class.

Output from agrVATE (included in the Bactopia pipeline) was used to determine *agr* type and detect any disruptive mutations in the *agr* operon (16). All assembled genomes from Patient 1 were identified as *agr* group 1, with no frameshifts detected. All assembled genomes from Patient 2 were identified as group 2, with no frameshifts in the *agr* operon. Isolates 3_111_SC through 3_116_SC from Patient 3 were classified as *agr* group 2 and had frameshifts present in their *agrC* reading frame; this finding is supported by their lack of hemolysis activity (Table 1)—a trait controlled by *agrC* (17). AgrVATE was able to identify multiple *agr* types present in isolate 3_110_Pool’s coding sequence—the pool sample for Patient 3—and it was ultimately classified as *agr* group 2. Isolates 3_117_SC and 3_118_SC from Patient 3 were identified as *agr* group 1, with no frameshifts in their *agrC* coding sequence (Table 2).

### Genomic comparisons with a previously sequenced *S. aureus* isolate

Our group had previously processed a sample taken from Patient 2 in February of 2012 (CFBR ID 196, Sample ID: CFBR_108; Dr. Eryn Bernardy), which was identified as MLST 225 and MRSA (2). Using the program snippy, we were able to estimate the number of SNPs between the 2012 isolate (Dr. Eryn Bernardy) and an isolate from the 2020 sample set, 2_102_SC, and we found 958 variants (18). Pairwise SNP distances across recent samples showed that the 2_105_MRSA sample has an average SNP distance of 657 to all single-colony isolates from Patient 2 but only 294 SNPs compared to the CFBR clinical isolate 2_CFBR, taken from the same sputum sample. 2_105_MRSA and 2_CFBR also share identical *agrC* sequences, with the same frameshift mutation present: c.878T > G p.Leu293*, resulting in an early stop codon.

### Further investigation of Patient 3 isolates

Based on initial findings of heterogeneity within the Patient 3 sample set, we elected to follow up with additional analysis of the antibiotic resistance profile among the pool and single-colony isolates (Table 3). The findings from Vitek 2 GP75 and *E*-test assays generally align with previous genomic indicators of resistance and susceptibility, with the exception that all isolates from this patient tested as susceptible to tetracycline—contradicting the finding of resistant alleles in all samples based on the AMRFinder Plus output. MRSA status is based on cefoxitin screen.

**TABLE 3:** Antibiotic resistance profile of Patient 3 isolates^*a*^^,^^*b*^

Isolate	Cefoxitin screen	MIC determined by Vitek 2 GP75/*E*-test if applicable												
		Oxacillin	Gentamicin	Ciprofloxacin	Levofloxacin	Moxifloxacin	Erythromycin	Clindamycin	Linezolid	Vancomycin	Doxycycline	Tetracycline	Trimethoprim- sulfamethoxazole (*E*-test only)	Rifampicin (*E*-test only)
3_110_Pool	**POS**	**≥4/24**	≤0.5	**≥8**	**≥8**	**≥8/>32**	**≥8**	**≥4/>256**	2/3	≤0.5/3	1/1.5	2/2	0.125	0.016
3_111_SC	**POS**	**≥4/3**	≤0.5	**≥8**	**≥8**	**≥8/>32**	**≥8**	**≥4/>256**	2/6	≤0.5/0.75	≤0.5/2	≤1/4	0.125	0.016
3_112_SC	**POS**	**1/1**	≤0.5	**≥8**	**≥8**	**≥8/>32**	**≥8**	**≥4/>256**	2/4	≤0.5/0.75	1/2	≤1/2	0.125	0.008
3_113_SC	**POS**	**≥4/**0.75	≤0.5	**≥8**	**≥8**	**≥8/>32**	**≥8**	**≥4/>256**	2/6	≤0.5/1	≤0.5/2	≤1/1.5	0.125	0.012
3_114_SC	**POS**	**≥4/1.5**	≤0.5	**≥8**	**≥8**	**≥8/>32**	**≥8**	**≥4/>256**	2/4	≤0.5/1	1/2	2/2	0.125	0.016
3_115_SC	**POS**	**0.5/0.75**	≤0.5	**≥8**	**≥8**	**≥8/>32**	**≥8**	**≥4/>256**	2/4	≤0.5/1	1/0.75	2/3	0.125	0.012
3_116_SC	**POS**	**≥4/12**	≤0.5	**≥8**	**≥8**	**≥8/>32**	**≥8**	**≥4/>256**	2/6	≤0.5/1	1/2	≤1/2	0.125	0.008
3_117_SC	NEG	≤0.25/1	≤0.5	≤0.5	≤0.12	0.047	≤0.25	0.094	2/4	1/1.5	≤0.5/0.25	≤1/0.38	0.125	0.012
3_118_SC	NEG	≤0.25/0.75	≤0.5	≤0.5	≤0.12	0.047	≤0.25	0.124	2/4	1/1.5	≤0.5/0.19	≤1/0.38	0.125	0.016

^*a*^ Bold text indicates resistance as determined by the MIC test.

^*b*^ MRSA is based on cefoxitin screen.

## DISCUSSION

### Sampling single colonies omits the true intraspecific diversity of CF *S. aureus* populations

Our initial hypothesis for this study was that by sampling both single colonies and pool samples of *S. aureus* populations isolated directly from freshly expectorated sputum, we could capture intraspecific diversity that may be missed by traditional clinical sampling methods. In support of this hypothesis, we found that the strain composition of two out of three archived clinical samples investigated was different compared to the majority of single colonies or pooled samples that were collected from their respective sputum samples. In the remaining patient sample (Patient 1), very little genetic diversity was detected among pooled, single colony samples, or archived clinical samples. We did, however, note important morphological diversity in this sample, as we initially observed small colony variants, although we were not able to further study these variants as they could not be recovered from the archived frozen stocks. Interestingly, we also found that in the case of samples from Patients 2 and 3, selection for methicillin-resistant (MRSA) isolates identified by the Emory Clinical Microbiology Laboratory masked *S. aureus* population diversity, as two of our pools contained both MRSA and MSSA strains (Table 1). Together, these findings suggest that both single isolates and pooled samples should be collected in order to capture the within-species *S. aureus* genetic diversity in a patient.

Specifically, we found that in the case of Patient 2, the population of MSSA (all Patient 2 isolates except for 2_105_MRSA) is likely the dominant lineage present in this sputum sample, which is not reflected in the archived MRSA clinical sample. In the case of Patient 3, an unsampled MSSA lineage was also identified by our sampling methods, which had not been recognized by the clinical microbiology laboratory (Table 1). While potentially not as numerically dominant as the MRSA population, these MSSA isolates are positive for hemolysis activity due to their functional *agrC*, while all MRSA isolates from Patient 3 have inactive *agrC*. This finding of a pathogen population that contains both a highly antibiotic-resistant lineage and a susceptible but potentially more virulent lineage demonstrates how complex *S. aureus* populations can be and highlights the potentially difficult task of finding a treatment approach that addresses both challenges. We also suspect that there is a third unsampled lineage present in Patient 3’s sample that was not detected by either the clinical lab or our approach of single colony and pooled sampling. This is based on the white colony morphology, which is present in the pool sample (3_100_Pool), but is not reflected in any single-colony isolate or the clinical sample. Altogether, these findings highlight observations that could be missed in research studies and clinical investigations that hope to link patient outcomes and the characteristics of clinical samples that may not reflect the true diversity of the pathogen population within an individual patient. We cannot recommend, however, shifting to only assessing pooled population samples, as antibiotic-resistant variants may remain unidentified by using this sampling method alone. Therefore, in line with other independent studies, we recommend the method of sampling a single colony along with a pooled sample from an initial selection plate as a useful additional approach for both clinicians and researchers to enable the identification of resistant variants, as well as an estimation of the overall diversity of a given pathogen population (4, 8).

### Diversity among single colony and pooled isolates yields insight into infection dynamics and pathogenicity

After our initial phenotypic analysis, we had concluded that the *S. aureus* isolates obtained from Patient 1 or Patient 2 were homogeneous and that the only within-patient diversity captured in our sample set was the two unique strains identified in Patient 3. However, after collecting more data on these samples, a more complex picture unfolded. It was interesting to see that the heterogeneity in Patient 3’s samples appeared in the genotypic analysis of 3_110_Pool as well, with multiple *agr* types having been identified by the *in silico* PCR program AgrVATE (Table 2). ANI analysis also allows us to distinguish the two lineages sampled from Patient 3 as truly distinct strains, based on the definition outlined in Rodriguez-R et al. (19), as the ANI values between isolates 3_111_SC through 3_116_SC are all at most only 99.00% similar to isolates 3_117_SC or 3_118_SC. Pairwise SNP distances also confirm that the two lineages in Patient 3 are unrelated, with more than 1,400 SNPs between all representative sample combinations. Additionally, we identified an unsampled lineage in our collection within Patient 2, as both pool samples, 2_100_Pool and 2_105_Pool, showed methicillin resistance by ChromAgar testing, while all single-colony isolates were MSSA (Table 2). Methicillin resistance was also noted by the Emory Clinical Microbiology Laboratory for the isolate (2_CFBR) from this sputum sample from Patient 2 (Table 1). A previous sample from Patient 2 from 2012, analyzed by our group, had also been identified as MRSA. Based on this previous isolate’s MLST (225) and the number of variants between 2_105_MRSA and a representative isolate from our 2020 sample set, 2_102_SC, we concluded that it is not part of the same lineage as the more recent isolates. In fact, the population isolated from the 2_105_Pool ChromAgar plate, 2_105_MRSA, also belongs to MLST (225) and is somewhat closely related to the clinical isolate 2_CFBR taken during the same sampling event, with less than 300 SNPs between the two samples. While this is more variation than we would expect to see within a single lineage over 8 years, it could be the case that the MLST 225 lineage had already been evolving within this host for some time and was already quite diverse at the time of sampling in 2012. These findings do indicate that there are multiple lineages coexisting within Patient 2 and that the MLST 225 population may have been evolving within this patient for over a decade. Overall, we were surprised at how much diversity we were able to capture with our limited patient sample set and simple isolate collection methods. To be able to apply these initial findings more broadly across pwCF or within-host *S. aureus* populations, larger studies that utilize a similar method of sampling individual colonies and pooled samples are required. The amount of clinically relevant diversity identified in our survey highlights the importance of bringing in ecologically minded sampling practices to patient sample surveys, especially in the case of long-term infections like those associated with CF.

### Population dynamics of co-occurring lineages

We postulate that our sampling methods have likely captured a shift in the frequency of the co-occurring lineages for Patients 2 and 3. In the case of Patient 3, it is likely that the MSSA population, which is not reflected in the clinical sample, is more virulent than the MRSA population due to its functional *agrC* and active hemolysis (17). Additionally, the dysfunctional *agrC* in the MRSA population in Patient 3 could signify its long-term presence in the host, as reduced *agr* activity is associated with persistence in chronic infections and immune evasion (20). It is interesting to have captured potentially dynamic periods within an ongoing infection. However, more broadly, our sampling methods and analysis of these lineages show that an ecological approach to sampling could yield important information about the genetic heterogeneity and changing population structure within a given sample and infection.

Future directions for this work include further testing and application of the sampling approach described here to a larger number of patient samples, as well as longitudinal samples from the same pwCF over time. Further investigation of the interactions and dynamics of co-occurring lineages in the presence of antibiotics is also warranted, with the opportunity to identify adaptive mutations in evolving populations. Recent studies that utilize similar sampling methods clearly demonstrate our ability to capture intraspecific diversity within individual patients and the compelling clinical implications it may have (4, 8). Additionally, when comparing findings of diversity within *S. aureus* populations to that of *P. aeruginosa*, we and others find that *S. aureus* populations frequently maintain multiple unrelated lineages, while *P. aeruginosa* populations show evidence of diversifying from a single lineage (4, 8). Importantly, for *S. aureus* populations where genes can be exchanged between unrelated variants, diversity within populations holds a high potential for enabling persistence in a host environment, as observed in previous studies (7). Taken together with the observations in this study, further investigation of pathogen population structures and their propensity to change over time within a host and in response to clinical treatment is highly justified. This initial study provides an exploratory but in-depth analysis of diversity across a small sample set. Importantly, we did not seek to replicate the host environment in our analysis of phenotypic or population analysis, and it is very likely that factors from the host environment and immune system would play a major role in changing the outcomes of intraspecific interactions in the host environment. Future studies should also include an inclusive set of pwCF and controls, including age range, coinfection status and history, and gender, for the best opportunity to link pathogen population diversity and individual health outcomes. Due to our limited sample set for this study, these were factors that could not be effectively assessed. Our findings presented here lay a foundation for a useful workflow and sampling approach that should be applied to a more extensive sample set to aid in answering highly pertinent questions related to the clinical treatment of chronic CF infections and within-host microbial ecology and evolution.

## MATERIALS AND METHODS

### Sample collection

The sampling methods used for the three sputum samples assessed in this study are outlined in Fig. 1. Fresh sputum was obtained from three different pwCF seen at the Emory Cystic Fibrosis Center; patient information and health status at the time of collection remained de-identified. These samples were spread on Mannitol Salt Agar (MSA; Remel) and incubated for 24–48 h of growth at 37°C. Eight single colonies from each sample were picked at random and inoculated into 3 mL of lysogeny broth (LB; Teknova). With the exception of Patient 1’s sample, morphological differences were not considered when selecting for single colonies. Patient 1’s sample had a significant number of small colony variants, and two small colonies were picked and archived but were unable to be revived from frozen stocks for further phenotypic or genotypic analysis. Liquid cultures were incubated overnight at 37°C in a rotating platform before being restruck on *Staphylococcus* Isolation Agar (SIA; BD Difco TSA with 7.5% NaCl) and made into 25% glycerol freezer stocks. All remaining colonies from the MSA plates were scraped together into a “pool” sample, which was resuspended in LB media and 25% glycerol and frozen directly. Samples 1_92_SC through 1_97_SC are single-colony isolates from Patient 1 (CFBR ID 623), and samples 1_90_Pool and 1_91_Pool are pool samples from the same patient. Samples 2_101_SC through 2_104_SC and 2_106_SC through 2_109_SC are single-colony isolates from Patient 2 (CFBR ID 196), and its pools from this patient are 2_100_Pool and 2_105_Pool. Samples 3_111_SC through 3_118_SC were single colonies isolated from Patient 3 (CFBR ID 311), and 3_110_SC is this patient’s pool sample (Table 2). Corresponding clinical isolates, 1_CFBR (Patient 1), 2_CFBR (Patient 2), and 3_CFBR (Patient 3), were collected, processed, and archived by the Emory Clinical Microbiology Laboratory and were made available to this study by the Emory Cystic Fibrosis Biospecimen Repository (CFBR) (Fig. 1).

### Clinical data collection

Antibiotic resistance testing results of *S. aureus* isolated from the Emory Clinical Microbiology Laboratory from Patients 1 and 2 (CFBR ID 623 and CFBR ID 196, respectively) were made available to us from the CFBR and are outlined in Table 1.

### Phenotyping

A qualitative analysis of colony morphology on tryptic soy agar was conducted on all isolates, including pool samples. While no significant differences in colony shape or structure were observed, colony color did vary across samples (white or yellow) and was recorded. While some small colonies were noted in Patient 1, we were unable to revive these variants after initial isolation. Alpha toxin production was observed by noting clear hemolysis on sheep blood agar plates. Congo Red agar (CRA) plates prepared as described by Freeman et al. (21) were used to determine polysaccharide production levels for all isolates by observing colony texture as previously described (2, 22). MRSA status was initially determined for all isolates by streaking each isolate and pool sample on ChromAgar MRSA plates and observing the presence or absence of purple pigmented colonies, indicating methicillin resistance (10).

### Whole-genome sequencing

To isolate genomic DNA for sequencing, all isolates and pool samples were streaked on SIA and incubated overnight at 37°C. The next day, cells were collected off plates with an inoculation loop (about a full loop), and resuspended in 480 µL of EDTA; 20 µL of 10 mg/mL lysozyme; and 20 µL of 5 mg/mL lysostaphin were then added to the resuspension, and mixtures were incubated at 37°C to lyse cells. After incubation, we proceeded with the rest of the protocol outlined for the Promega Wizard Genomic DNA Purification Kit. Sequencing was conducted using the Illumina NextSeq 2000 platform at the Microbial Genome Sequencing Center (Pittsburgh, PA, USA). Genome sequences were evaluated for quality using the program FASTQC (23). Genome sequences were made publicly available at the NCBI BioProject accession PRJNA742745. Samples listed in this BioProject will have the prefix CFBR_EB_Sa before their sample number Sa###; numbers in this BioProject correspond to the same sample number in this study.

### Analysis of genomic diversity

Raw fastq files (a total of 30 genomes) were run through the Bactopia genome analysis pipeline version 3.0.1 with a maximum of four CPUs specified; otherwise, default parameters were used to obtain assemblies for all genomes (3). Using these assemblies, ARIBA was used to determine MLSTs, and NCBI AMRFinderPlus version 3.12.8 was used to evaluate antimicrobial resistance-associated genotypes (15, 24) (Fig. 3). AgrVATE version 1.0.2 was used to determine the *agr* type and look for the presence of frameshift mutations for all samples (https://github.com/VishnuRaghuram94/AgrVATE) (16).

Population-level genomic analysis was conducted by building a core genome alignment with Parsnp version v2.0.5 using USA300 JE2 NCBI accession NC_007793 as a reference (12, 13). The genome alignment was then processed with Disty McMatrixface software v0.1.0 to create a distance matrix of single-nucleotide polymorphisms (SNPs) (https://github.com/c2-d2/disty). This file was then converted into a neighbor-joining tree rooted at the midpoint of the sample set using the R packages ape v5.0 and phangorn v2.12.1 and then visualized with ggtree v3.12.0 (Fig. 2A) (14, 25, 26). The program pyANI v0.2.12 was used to calculate average nucleotide identity (ANI) within patient samples and across all samples (27). The R package pheatmap v1.0.12 was used to generate a heatmap of sample ANI values (Fig. 2B) (28). To further illustrate the relatedness of samples within each patient set, SNP distance heatmaps were generated using the R package pheatmap v1.0.12 for the entire sample set and each patient sample set (Fig. 2C). To compare the relatedness of a pair of longitudinal samples, the program Snippy v4.6.0 (18) (https://github.com/tseemann/snippy) was used to estimate the number of single-nucleotide polymorphisms between a previous isolate from Patient 2 in 2012 (CFBR_108) and the isolates obtained in 2020 for this study (2).

### Antimicrobial susceptibility testing

Automated antimicrobial susceptibility testing was performed on a Vitek 2 compact instrument using AST-GP75 cards following the manufacturer’s instructions (bioMérieux, Inc Durham, NC, USA). Liofilchem MIC Test Strips (MTS) were used as a quantitative assay for determining the minimum inhibitory concentration (MIC) according to the MTS Application Guide (Liofilchem, Roseto degli Abruzzi, Italy). Briefly, a 0.5 McFarland turbidity standard, corresponding to approximately 1.5 × 10^8^ CFU/mL was prepared in 0.45% saline and spread onto Mueller Hinton II agar (Hardy Diagnostics, Santa Maria, CA, USA). A Liofilchem MTS was added, and the plates were incubated at 35°C for 24 h in ambient air. The MIC was recorded, and susceptibility results were determined using Clinical and Laboratory Standards Institute (CLSI) guidelines (29).
